# Quaternary ammonium compounds in hypersensitivity reactions

**DOI:** 10.3389/ftox.2022.973680

**Published:** 2022-09-16

**Authors:** Marine Peyneau, Luc de Chaisemartin, Nicolas Gigant, Sylvie Chollet-Martin, Saadia Kerdine-Römer

**Affiliations:** ^1^ Université Paris-Saclay, Inserm, Inflammation microbiome immunosurveillance, Châtenay-Malabry, France,; ^2^ Department « Autoimmunité, Hypersensibilités et Biothérapies », DMU BioGeM, APHP, Hôpital Bichat, Paris, France; ^3^ CNRS, BioCIS, Université Paris-Saclay, Châtenay-Malabry, France

**Keywords:** quaternary ammonium, hypersensitivity, sensitization, disinfectant, antibacterial, irritant, sensitizer, adjuvant

## Abstract

Quaternary ammonium compounds (QAC) are commonly used disinfectants, antiseptics, preservatives, and detergents due to their antibacterial property and represent the first used biocides before phenolic or nitrogen products. Their common structure consists of one or more quaternary ammonium bound with four lateral substituents. Their amphiphilic structure allows them to intercalate into microorganism surfaces which induces an unstable and porous membrane that explains their antimicrobial activity towards bacteria, fungi, and viruses. QAC are thus found in many areas, such as household products, medicines, hygiene products, cosmetics, agriculture, or industrial products but are also used in medical practice as disinfectants and antiseptics and in health care facilities where they are used for cleaning floors and walls. QAC exposure has already been involved in occupational asthma in healthcare workers or professional cleaners by many authors. They also have been suggested to play a role in contact dermatitis (CD) and urticaria in workers using cosmetics such as hairdressers or healthcare workers, inciting reglementary agencies to make recommendations regarding those products. However, distinguishing the irritant or sensitizing properties of chemicals is complex and as a result, the sensitizing property of QAC is still controverted. Moreover, the precise mechanisms underlying the possible sensitization effect are still under investigation, and to date, only a few studies have documented an immunological mechanism. Besides, QAC have been suggested to be responsible for neuromuscular blocking agents (NMBA) sensitization by cross-reactivity. This hypothesis is supported by a higher prevalence of quaternary ammonium (QA)-specific IgE in the professionally exposed populations, such as hairdressers, cleaners, or healthcare workers, suggesting that the sensitization happens with structurally similar compounds present in the environment. This review summarizes the newest knowledge about QAC and their role in hypersensitivities. After describing the different QAC, their structure and use, the most relevant studies about the effects of QAC on the immune system will be reviewed and discussed.

## 1 Introduction

Quaternary ammonium compounds, commonly named QAC or quats, have been used for their antimicrobial property since the beginning of the 20th century (Jacobs et al., 1916a, Jacobs et al., 1916b; Jacobs, 1916). QAC have the general structure of a positively charged nitrogen atom, bound to four carbon atoms, and a negatively charged anion, which is biologically inactive. In 1916, Jacob and colleagues discovered the germicidal activity of quaternary salts of hexamethylenetetramine and derivates by investigating the relation between the chemical structure and the bactericidal activity (Jacobs et al., 1916a, Jacobs et al., 1916b; Jacobs, 1916). A few years later, the German Nobel prized Gerhard Domagk realized the potentiality of QAC with the discovery of the well-known and currently widespread benzalkonium, or BAC, in 1935 (Domagk, 1935). He found that if at least one of the groups bound to the QA was a long alkyl chain, the molecule could kill micro-organisms. Indeed, the positive head of the molecule adheres to the negatively charged surface of micro-organisms (bacteria, virus), and the long alkyl chain inserts into the lipid membrane rendering it porous and causing cellular content to leak out. BAC was the first QAC approved by the United States Environmental Protection Agency in 1947 (US EPA, 2006). Since then, different structures of QAC have been described, among which the trimethylalkylammonium based-QAC, from which cetyltrimethylammonium bromide or CTAB is the most famous, or the dialkyldimethylammonium based-QAC, whose main component is the dimethyldidecylammonium chloride or DDAC (Table 1).

**TABLE 1:** Classification of quaternary ammonium compounds and their main uses.

Types of QAC		General structure	Members of the group	CAS number	Use
Mono-QAC	Tetraethylammonium TEA	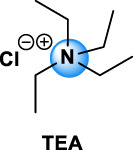	TEA	71-91-0	Potassium channel blocker Furman, (2018)
	Trimethylalkylammonium based-QAC: HTA type	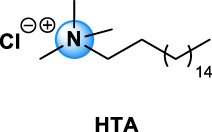	HTA, CTAB	HTA: 112-02-7	Polish, lacquers, sanitizers and road construction materials Nielsen et al. (2007)
				CTAB: 57-09-0	
	Dialkyldimethylammonium based-QAC: DDA-type	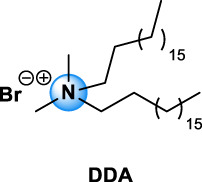	DDA, DDAC	DDA: 3700-67-2	Disinfecting/detergent in hospital, antimicrobial agent (i.e. wood treatment), algicides in swimming pools, fungicide, fabric refreshers and softeners, paint and polish. Adjuvant in veterinary and experimental vaccines Hilgers and Snippe, (1992), Nielsen et al. (2007)
				DDAC: 7173-51-5	
	Pyridinium based-QAC: CPC type	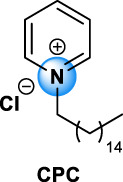	CPC	6004-24-6	Preservative in pharmaceutical use: oral care products (toothpaste, mouthwash), nasal sprays, throat lozenges Mao et al. (2020)
	BAC-type	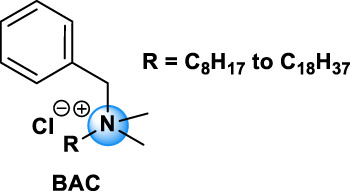	BAC, BE, CKC, miramistin	BAC: 63449-41-2	Fabric softeners, personal hygiene and cosmetic products, preservative in eye drop and medications, disinfectants (household, agriculture, industrial, clinical settings) Merchel Piovesan Pereira and Tagkopoulos, (2019)
				BE: 121-54-0	
				CKC: 122-18-9	
PolyQAC		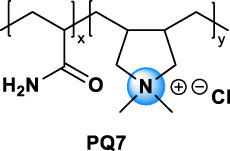	PQ7, PQ10…	PQ7: 26590-05-6^a^	Antistatic, softener used in cosmetics (shampoo, conditioner, shower gel, make-up.) Andersen, (2011)
				PQ10: 68610-92-4^a^	
Bis-QAC	OCT	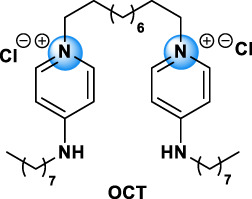		70775-75-6	Skin and wounds antisepsis, disinfectant, disinfection of surgical equipment Vereshchagin et al. (2021)
	Diquat	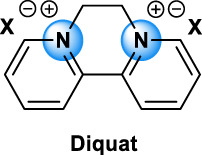		^a^	Herbicides Akhavein and Linscott, (1968)
	Paraquat	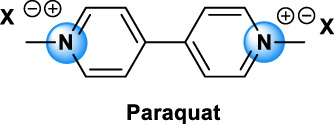		^a^	Herbicides Akhavein and Linscott, (1968)

TEA, tetraethylammonium chloride; HTA, hexadecyltrimethylammonium chloride; CTAB, cetyltrimethylammonium bromide; DDA, dimethyldioctadecylammonium bromide; DDAC, dimethyldidecylammonium chloride; CPC, cetylpyridinium chloride; BAC, benzalkonium chloride; BE, benzethonium chloride; CKC, cetalkonium chloride; OCT, octenidine dihydrochloride; PQ7, polyquaternium-7; PQ10, polyquaternium-10.

a Different CAS numbers for these compounds or group of compounds.

QAC are the first used biocides before nitrogen-containing scaffolds, phenolic compounds and aldehydes (Vereshchagin et al., 2021). They represent a broad class of molecules whose market was estimated at $963.7 million in 2019 (XiaoZhi, 2020). They are used in various areas such as the medical field (disinfection of surgical and medical instruments, cleaning the surfaces…), industry, agriculture, or household. Considering their intrinsic properties (antimicrobial, antistatic, film-forming, or preservative), they can be found in numerous products such as cleaning products, herbicides, disinfecting products (wipes, soaps, sprays…), cosmetics (shampoo, shower gel, toothpaste…), eye drops, pharmaceutical products (throat lozenges, nasal spray…), which make them widespread use products.

QAC have been reported in immediate hypersensitivity reactions (asthma, contact urticaria) (Bernstein et al., 1994; Burge and Richardson, 1994; Purohit et al., 2000; Houtappel et al., 2008; Oropeza et al., 2011) but also delayed reactions (CD, eczema) (Klein et al., 1991; Cusano and Luciano, 1993; Dejobert et al., 1997; Mauleón et al., 2006; Ulicki et al., 2022) in the exposed populations. They also have been suggested to be the sensitizing molecules at the origin of NMBA anaphylaxis (Florvaag et al., 2005; de Pater et al., 2017). The underlying mechanisms are still controverted, as QAC have been described as irritants, adjuvants, or sensitizers, depending on the studies. This review firstly aims to describe the different QAC currently used and the relationships between their structure and use. Then, we will focus on the different reports of their implication in human health, with a deep study on the immunological mechanisms involved.

## 2 Quaternary ammonium compounds: Structures and link to their biocide activity and use

### 2.1 The structure of quaternary ammonium compounds

The structure of QAC consists of a positively charged nitrogen atom bound to three or four substituents, and a negatively charged anion, either a bromide or a chloride. Both anions have no biological function but enable the solubility of the compound.

**FIGURE 1:**
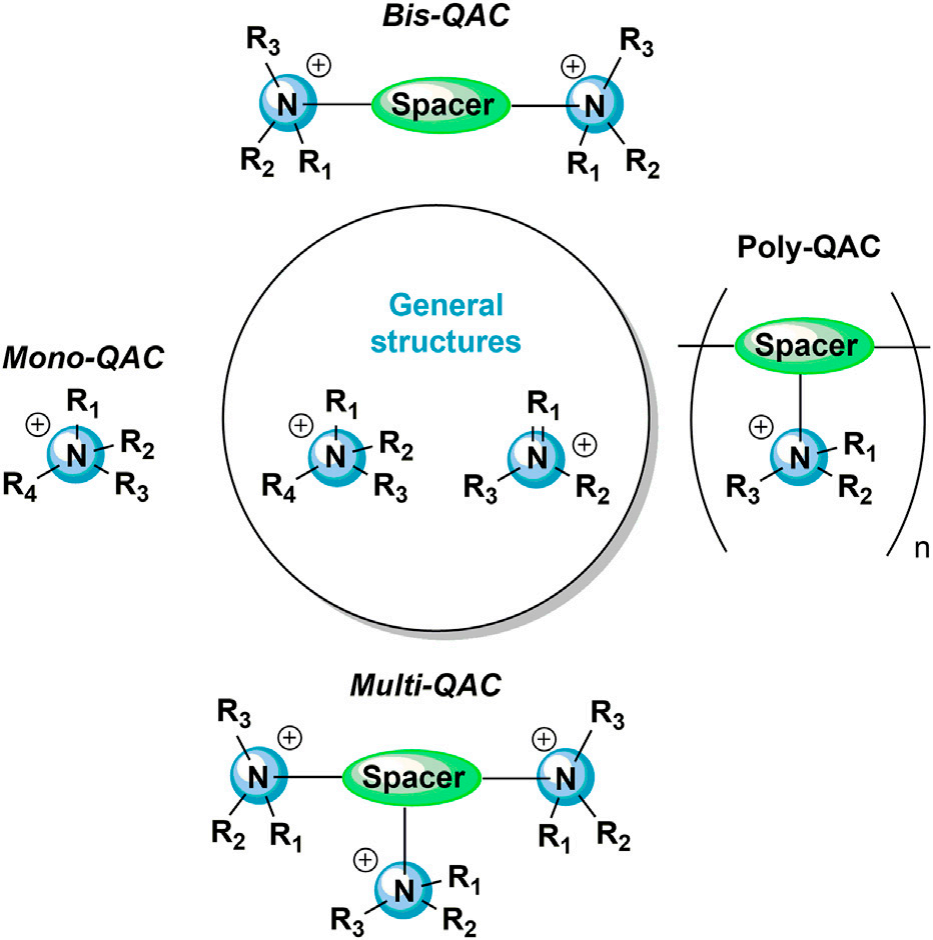
The general structure of QAC.

The different types of QAC can be classified according to the number of positively charged nitrogens in their structure and, more precisely, according to lateral chains (Vereshchagin et al., 2021). The first classification distinguishes mono-QAC (one nitrogen), bis-QAC (two nitrogens), or the so-called twin surfactant, multi-QAC, and poly-QAC (more than two nitrogens). Multi-QAC represent molecules that contain at least three ammoniums, each of them being linked to a pattern called “spacer.” In contrast, poly-QAC or polyquaterniums are polymers of high molecular weight that contain an increased number of a repeated pattern, including a nitrogen (e.g., polyquaternium-7 or PQ7, CAS No: 26590-05-6). Each polyquaternium is followed by a number assigned in the order of their registration by the International Nomenclature for Cosmetic Ingredients (INCI) (Figure 1).

The different types of QAC are summarized in Table 1 according to this classification, (Larsen et al., 2004a; Vereshchagin et al., 2021).

### 2.2 Quaternary ammonium compounds mechanism of action and relationships between their structure and their activity

The surface of bacterial cells universally carries a negative charge stabilized by divalent cations such as Mg^2+^ or Ca^2+^. The cell phospholipid membrane is covered with peptidoglycan containing teichoic acid and polysaccharide elements in Gram-positive bacteria. In contrast, in Gram-negative bacteria, the peptidoglycan is covered with another layer of lipopolysaccharide (Gilbert and Moore, 2005). Each surface also contains proteins necessary for the structural integrity of the membrane, catabolism, cellular transport, or biosynthesis of extracellular products (e.g., toxins) (Gilbert and Moore, 2005). When a QAC makes contact with a cell membrane, the positively charged nitrogen strongly binds to the bacteria’s negatively charged membrane. While the positive head of the molecule stays outside the membrane, the hydrophobic lateral chain becomes immersed into the phospholipid layer. This intercalation induces a perturbation of the phospholipid bilayer membrane, an alteration of the configuration of membrane proteins, and a leakage of the cytoplasmic content out of the bacteria leading to its death (Gilbert and Moore, 2005). This activity can be maximized according to the lateral chain length of the QAC: for Gram-positive bacteria, this activity is maximized with chain lengths shorter than 12–14 carbons, whereas for Gram-negative bacteria, the optimal activity is conferred by compounds with a lateral chain of 14–16 carbons (Gilbert and Moore, 2005). For example, considering BAC or HTA-type (hexadecyltrimethylammonium chloride) QAC, the compounds containing 14 or 16 carbons exhibit the strongest antibacterial activity (Gilbert and Al-taae, 1985; De Saint Jean et al., 1999).

Moreover, the addition of a second long aliphatic chain increases the anti-bacterial effect (Jennings et al., 2015). There are many explanations for this phenomenon. First, QAC can form micelles due to their amphiphilic structure. This possibility increases the antibacterial effect of QAC (Zhou and Wang, 2020). Micelle formation has been shown to be easier for longer compounds with hydrophobic chains than for shorter compounds, since it requires some distance between the hydrophobic part and the positively charged nitrogen. Second, there might be a different affinity for the surface of the bacteria depending on the length of the compound. Third, according to the type of microorganisms, QAC do not exhibit the same activity (mainly because of the difference in the composition of the envelope). Concerning bacteria, this is mainly due to the thickness of the membrane of the Gram-negative bacteria, which harbour two layers of membrane. QAC exhibit a stronger activity on Gram-positive bacteria than on Gram-negative (Jennings et al., 2015). Thus, mixtures of different lengths QAC are being used to widen their spectrum of affinity. More details about the relationships between the structure of QAC and their antibacterial activity are available in the excellent reviews of Jennings et al., 2015 and Vereshchagin et al., 2021.

### 2.3 The widespread use of quaternary ammonium compounds

QAC exhibit numerous physicochemical properties rendering their use widespread. First, QAC are extremely powerful biocides. They are used as pesticides (herbicides or algicides) in the agriculture area, disinfectants, detergents, and sanitizers (household or institutional), preservatives (contact lens cleaning solutions, aerosolized asthma medications, body care products (soap, skin ointments…)), or water process treatments (Vereshchagin et al., 2021). QAC are the main components of cleaning products.

QAC also have non-antimicrobial properties, especially due to their amphiphilic structure: antistatic (surfactant, tensioactive) and softener. They are thus found in various cosmetic products (shampoo, conditioners, dyes, and hygiene products such as toothpaste). For example, PQ7 was approved by the Food and Drug Administration in 1995 and is now widely used in nearly thousands of products such as shampoos, conditioners, shower gel, eye makeup, hair dyes shaving products, etc. (Andersen, 2011). QAC are also found in drugs, such as NMBA. The QA moiety has been found to be responsible for the pharmacological properties of NMBA (Baldo and Fisher, 1983a). The principal applications of QAC are summarized in Table 1.

## 3 Quaternary ammonium compounds-associated immediate hypersensitivity reactions

Several authors have incriminated QAC in the onset of some immediate hypersensitivity reactions. Those reactions occur within a few minutes after the exposure to an allergen. They are classically mediated by an IgE-dependent immune response. Symptoms, like asthma, urticaria, dermatitis, conjunctivitis, or anaphylaxis, result from histamine release by basophils and mast cells. Symptoms of asthma have been reported following BAC occupational exposition in factory workers, pharmacists or nurses (Bernstein et al., 1994; Burge and Richardson, 1994; Purohit et al., 2000). In those case reports, patients had no QA-specific IgE but experienced pulmonary symptoms during the re-challenge, confirming the incrimination of BAC (Bernstein et al., 1994; Burge and Richardson, 1994; Purohit et al., 2000). BAC used as a preservative in asthma medicine Atrovent^®^ has also been reported to induce paradoxical and severe bronchoconstriction (Beasley et al., 1987). DDAC was also responsible for contact urticaria in a laboratory technician (Oropeza et al., 2011). Another QAC was reported in immediate reaction (urticarial eruption and respiratory discomfort) in an anaesthetic nurse a few minutes after using a disinfectant product for an endoscope containing *N, N*-didecyl-*N*-methyl-poly(oxyethyl) ammonium propionate. The diagnosis was confirmed by performing prick-test (Raison-Peyron et al., 2009). In those case reports, QAC are clearly incriminated, but sometimes, QAC responsibility is only suggested, and definite proof is lacking (Rosenman et al., 2003). To better characterize the effect of cleaning products on pulmonary functions, Vandenplas et al. (2013) realized a retrospective study of workers with suspected occupational asthma. Workers were explored by specific inhalation challenge (SIC), bronchial hyperresponsiveness to histamine, and sputum cell count. Their results showed that 10/17 were exposed to QAC (mainly BAC and DDAC) among the positive SIC in the patients (Vandenplas et al., 2013). Noticeably, among the participants who developed a positive bronchial response to QAC, a post-challenge increase in sputum eosinophils and/or in the level of airway hyperresponsiveness was documented in 9 of 10 instances which was according the authors, in favor of a specific reaction (Vandenplas et al., 2013). Importantly, anaphylaxis was also reported after BAC exposure contained in nose drops (Mezger et al., 2012) and in eye drops (Anderson et al., 2009). Another anaphylactic reaction happened during a skin prick test with BAC, in the exploration of a suspected allergy to the nebulizer solution used to treat asthma (Kim and Ahn, 2004).

Apart from those case reports and SIC investigations, epidemiological studies have been performed to study the link between hypersensitivity reactions and the exposure to QAC. For example, Vogelzang et al. (1997) realized a cross-sectional study of pig farmers and showed that bronchial hyperresponsiveness to histamine, a hallmark of asthma, was detected in farmers using QAC as disinfectants for animal cleaning, especially if QAC were the principal active components of the disinfectants. However, those results were not found in a 3-year follow-up study by the same author (Vogelzang et al., 2000).

Occupational asthma is a significant concern for hospital workers and cleaners widely exposed to QAC. As a result, epidemiological studies are linking exposure in these workers to the development of asthma. For example, a large cohort of American nurses was studied for asthma control regarding their occupational exposure. In this study, the authors found no association between QAC exposure and asthma control (Dumas et al., 2017). In contrast, another French study of healthcare workers showed a strong association between the exposure to QAC and asthma or nasal symptoms (Gonzalez et al., 2014). Surveillance networks of occupational health can also provide information about the role of QAC in the onset or the exacerbation of asthma. In this context, Paris et al. showed an increase in occupational asthma related to QAC exposure in France in 2001–2009, although overall work-related asthma tends to decrease over time (Paris et al., 2012).

However, studies that explore occupational asthma in cleaners do not always precise the composition of the products responsible for respiratory symptoms so that quaternary ammonium are not specifically incriminated (Medina-Ramón et al., 2006, 2005; Bernstein et al., 2009), even if QAC are known to be the main component of cleaning agents. Lakind and Goodman recently published an excellent review about occupational asthma and QAC (LaKind and Goodman, 2019).

As already described earlier, immediate hypersensitivity symptoms are the results of histamine release. BAC has been shown to induce the release of histamine by mastocytes *in vitro*, and to inhibit the 48/80 compound-, NMBA-, and substance P-induced histamine release (Read and Kiefer, 1979). The latter compounds are three agonists of the recently discovered Mas-related G-protein-coupled receptor member X2 (MRGPRX2) (McNeil et al., 2015), but further studies will be necessary to understand the underlying mechanisms. However, this direct histamine release was suspected in asthmatic patients as histamine antagonists were partly able to inhibit BAC induced-bronchoconstriction (Miszkiel et al., 1988b).

## 4 Quaternary ammonium compounds-associated delayed hypersensitivity reactions

Delayed hypersensitivity reactions to QAC have also been described. Those reactions generally occur within 24–72 h after allergen exposure, and the classical mechanism involves macrophages, specific T lymphocytes, and interferon γ (IFN-γ) release. Symptoms are caused by inflammatory cytokines released by IFN-γ activated macrophages and cytotoxic T cells that can mediate direct cytotoxicity (Pichler, 2003). Symptoms of delayed hypersensitivity range from mild reactions such as eczema and CD, to severe systemic reactions (i.e., DRESS syndrome or Steven-Johnson syndrome).

CD to QAC has been well described in healthcare workers (HCW) and cleaners. For example, BAC has already been reported in CD in a nurse which used BAC in sterilizing solution (Cusano and Luciano, 1993) and in surface disinfectants (Klein et al., 1991). Airborne CD was also described in a cleaning woman due to a spreading on the floor of QUAT-10^®^ containing BAC (Mauleón et al., 2006). In a hospital cleaner, BAC and DDAC were also recently incriminated in severe dorsal and palmar hand dermatitis (Ulicki et al., 2022). DDAC had already been described in other case reports of CD in hospital workers (Dejobert et al., 1997; Dibo and Brasch, 2001; Geier et al., 2013). CD to PQ7 was also described in a nurse with a history of work-related eczema of the hands spreading to the arms, face, and neck (Gallo et al., 2001). Another QAC, *N, N*-didecyl-*N*-methyl-poly(oxyethyl) ammonium propionate, was reported in CD in a dental assistant (De Quintana Sancho et al., 2014).

Cohorts of HCW and non-HCW with CD were compared to provide information on the origins of sensitization. Three studies found a significantly higher incidence of CD to BAC in HCW than in non-HCW (Shaffer and Belsito, 2000; Nettis et al., 2002; Suneja and Belsito, 2008). The prevalence of BAC sensitization in HCW with CD ranges from 7.1% to 9.7% (Shaffer and Belsito, 2000; Nettis et al., 2002; Suneja and Belsito, 2008). Concerning quaternium-15 Suneja and Belsito found a significantly higher rate of sensitization in HCW than in non-HCW (34% vs. 13.7%, *p*< 0.001), but it was not found in an older study of the same researchers (Shaffer and Belsito, 2000).

Contact allergies were also described with QAC contained in topical medicinal products. For example, contact allergy was found to be common in patients using BAC-containing topical medicine for chronic leg ulcers or stasis dermatitis, with a sensitization prevalence ranging from 7% to 18.6% (Saap et al., 2004; Barbaud et al., 2009; Valois et al., 2015; Raudonis et al., 2019; Silverberg et al., 2021). Another condition, flexural eczema (eczema affecting the skin where the joint bends with low pruritic lesions) and irritant dermatitis were also reported after the use of BAC in antiseptic bath oil at high concentrations (Ling and Highet, 2000; Loo et al., 2003; Storer et al., 2004; Hann et al., 2007). The fact that these patients present alteration of the skin barrier could constitute a risk factor for sensitization by facilitating the cutaneous penetration of QAC.

BAC and benzethonium (BE) are also used as preservatives in medicines. Using patch testing, sensitization to BAC and/or BE were found in patients with contact allergies to ear drops (Fräki et al., 1985), nose drops (Lechien et al., 2018), eye drops (Fisher and Stillman, 1972; Afzelius and Thulin, 1979; Aoki, 1997), and inhaled or intranasal corticosteroids (Bennett et al., 2001). The diagnosis in those cases might be difficult as symptoms of mucosal contact hypersensitivity can be mild and may appear only as a failure to the treatment or a worsening of the underlying condition (asthma, conjunctivitis, allergy…).

CD has also been described in patients using BAC and quaternium-15 contained in cosmetics (Amin and Belsito, 2006; Dastychová et al., 2008). Moreover, a significant association was found between eyelid dermatitis and sensitization to BAC (Amin and Belsito, 2006). A series of six patients with eyelid dermatitis to shellac (insect-derived texture agent) contained in mascaras revealed the co-sensitization to quaternium-22 in one patient. Quaternium-22 is used as a film-former, antistatic, and hair-conditioning agent, mainly in mascaras, hair conditioners, soaps, shampoos, and detergents (Le Coz et al., 2002). This co-sensitization has already been described with mascara (Scheman, 1998). More anecdotally, DDAC was incriminated in foot dermatitis by using a shoe refresher spray (Mowitz and Pontén, 2015).

BAC has been the most reported QAC in the literature for contact allergies. This is probably because it is the most widespread QAC as preservative and antiseptic in medicines, cosmetics, and cleaning agents, but it might also be due to its intrinsic properties. A study of patients with contact allergy and CD found a trend to increase in BAC sensitization in the recent years, and the main sources of exposure were ophthalmic drops, topical antiseptics, and cosmetics (Dear et al., 2021). Moreover, BAC was the sixth allergen giving the highest reaction within a large retrospective cohort of patch testing (Veverka et al., 2018).

## 5 Understanding the underlying mechanisms of quaternary ammonium compounds-induced hypersensitivities: Between irritant, sensitizer, and adjuvant

### 5.1 Irritancy potential

**FIGURE 2:**
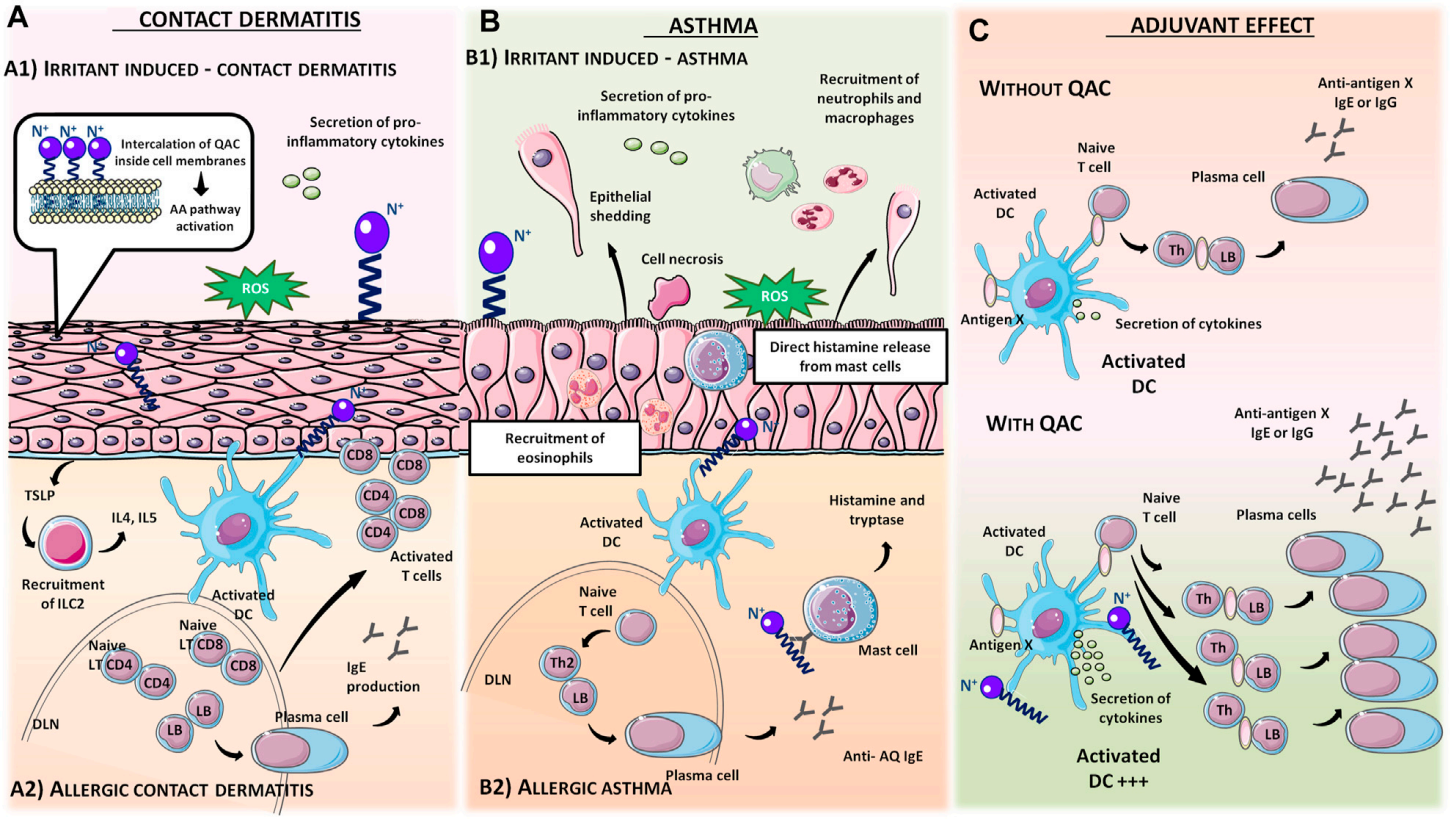
Putative mechanisms of action of QAC. According to the literature, QAC have been reported in contact dermatitis **(A)** and in asthma **(B)**. They own irritant properties **(A1,B1)**, but have also been described as sensitizers **(A2,B2)**. Other reports attribute adjuvant effect to QAC **(C)**. **(A1)** In the irritant induced-contact dermatitis mechanism, QAC alter the skin by the production of ROS and the activation of the AA pathway. **(A2)** QAC can also activate the immune system in allergic contact dermatitis, by triggering a specific cellular response, with the recruitment of CD4^+^ and CD8^+^ T cells in the skin. ILC2 have also been reported in the pathophysiology and anti-QA specific IgE have also been described in response to QAC applied on the skin. **(B1)** Asthma symptoms of QAC can be attributed to irritant mechanisms, by the production of ROS, epithelial shedding, cell necrosis, the recruitment of neutrophils and macrophages and the release of proinflammatory cytokines. Direct histamine release has also been described. **(B2)** Allergic asthma is mediated by a specific immune response, mediated by Th2 lymphocytes, leading to the production of anti-QA specific IgE, the degranulation of mast cells and the recruitment of eosinophils. **(C)**. The adjuvant effect is described as the increase of the allergenicity of other compounds. AA, arachidonic acid; DC, dendritic cell; DLN, draining lymph node; ILC2, type-2 innate lymphoid cells; LB, B lymphocytes; LT, T lymphocytes; QA, quaternary ammonium; QAC, quaternary ammonium compound; ROS, reactive oxygen species; Th, helper T cell; TSLP, thymic stromal lymphopoietin.

A definition from the US Occupational Safety and Health Association states: “Irritants are noncorrosive substances that cause temporary inflammation on direct contact with the skin, eyes, nose, or respiratory system by a chemical action at the point of contact” (The OSHA hazard communication standard, 1994). The action of irritants do not entail an immunologic mechanism. The irritancy of a chemical is linked to its ability to damage the surface lipid layer and/or induce cellular damage. Given that QAC antibacterial effect acts through lipid layer disturbance, it is therefore possible that QAC own irritant properties (Figures 2A1,B1).

#### 5.1.1 Irritant-induced asthma due to quaternary ammonium compounds exposure

Different mechanisms underlying asthma development have been proposed (Tarlo and Lemiere, 2014). Asthma can be either sensitizer-induced, with an IgE or a non-IgE dependent pathway, or irritant-induced (Figure 2B). Clinically, those different pathways are difficult to differentiate, and QAC have been reported to induce both types of asthma (Quirce and Barranco, 2010; Burge et al., 2012).

Inhalation of irritants induces bronchial epithelial damage, which results in proinflammatory responses, neurogenic inflammation, increased lung permeability, and the remodeling of the airway epithelium. Histological findings of irritant-induced asthma described epithelial cellular injury and bronchial wall inflammation (Tarlo, 2013; Tarlo and Lemiere, 2014). The sputum generally exhibits low or no eosinophilia but rather a neutrophilic infiltration, and in the case of high-level exposure to an irritant, bronchial biopsies show intense epithelial shedding (Tarlo and Lemiere, 2014; Vandenplas et al., 2014; Esteban-Gorgojo et al., 2018). The pro-inflammatory response might result from an inappropriate oxidative stress caused by irritant-induced epithelial damage (Tarlo and Lemiere, 2014), even if the pathogenic mechanism is still largely unknown.

Some animal exposure studies have been carried out to elucidate the pathogenic mechanisms of QAC on pulmonary functions. Larsen et al. (2012) showed the irritancy effect of different QAC (BAC, HTA, CPC (cetylpyridinium chloride), and DDAC) by inhalation in mice. Animals experienced a dose-dependent decrease in the tidal volume and a concomitant increase in respiratory rate; moreover, BAC was the most potent among the QAC tested. They also showed an acute pulmonary inflammation with infiltration of neutrophils and macrophages in the bronchial alveolar lavage fluid (BALF). Those effects were independent of trigeminal nerve stimulation, histamine H1 receptors or muscarinic receptors.

Furthermore, Swiercz showed that BAC exposure produced a strong pneumotoxic effect evidenced by respiratory cells necrosis and lung inflammation with an increase in interleukin (IL)-6 level in BALF (Swiercz et al., 2008). A 2-week exposure of BAC by inhalation also showed the irritative effect of BAC on rats. Animals exposed to the highest dose of 20 mg/m^3^ showed deep breathing, hoarseness, and nasal discharge, and surprisingly a decrease in reactive oxygen and nitrogen species in IL-1β, IL-6, and macrophage inflammatory protein (MIP)-2 in BALF, which were accompanied by cellular changes in the lungs and the nasal cavity tissues such as hypertrophy, hyperplasia, metaplasia or necrosis (Choi et al., 2020). Intratracheal instillation of DDAC at 150 μg/kg for 7 days in mice were also shown to induce alveolar cell injuries, the recruitment of pro-inflammatory cells, the release of IL-6, MIP-1α, RANTES (Regulated upon Activation Normal T cell expressed and secreted), monocyte chemoattractant protein (MCP)-1, and MIP-1β in BALF (Ohnuma et al., 2011, Ohnuma et al., 2010) and to cause fibrosis in the lung (Ohnuma-Koyama et al., 2013). However, in the studies of Lim and Chung (2014), rats exposed to DDAC for 2 weeks or 13 weeks by inhalation at three different concentrations showed only little inflammatory cell infiltration and no fibrosis, but only a weight loss for the higher concentration (Kim et al., 2017). CPC was also shown to induce a cytotoxic effect *in vitro* on A549 cells and *in vivo* on rats. Intratracheal instillation of CPC on rats induced pulmonary cell injuries, the recruitment of neutrophils in BALF, and the release of proinflammatory cytokines, of which tumor necrosis factor (TNF)-α was the most importantly secreted (Kim et al., 2021).

*In vitro* analysis of BAC and CPC on A549 cells showed that those QAC induced a dose-dependent apoptosis of the cells by activating the caspase-3 dependent pathway and altered the alveolar surfactant activity. Those effects were attributed to the long alkyl chain of the QAC, rather than to the hydrophilic quaternary ammonium pattern (Kanno et al., 2020). Moreover, Kwon et al. (2014) showed that DDAC induced reactive oxygen species (ROS) production and decreased glutathione activity on lung epithelial cells. As explained earlier, oxidative stress might be responsible for the pro-inflammatory effects observed in response to QAC (Tarlo and Lemiere, 2014). However, some authors also point to a potential role of mast cell activation (Miszkiel et al., 1988a) (Figure 2B1).

Some clinical studies or case reports favor an irritative mechanism of QAC (Balmes, 2002; Quirce and Barranco, 2010; Gonzalez et al., 2014). Indeed, QAC have been reported to induce asthma from the first exposure, which is in contradiction with the sensitization needed in an IgE-dependent asthma. Moreover, in some studies, QAC do not appear as strong specific IgE inducers. Furthermore, some results seem to underline the irritative effect of QAC in the onset of respiratory symptoms, especially when QAC induce the exacerbation of a preexisting asthma (Sly, 1990; Zhang et al., 1990).

#### 5.1.2 Irritant-induced dermatitis due to quaternary ammonium compounds exposure

As for asthma, QAC-induced dermatitis can be due to either an irritative or a sensitizing mechanism (Figure 2A). Clinically, the symptoms are very similar. Skin irritation and the associated inflammatory response involve interactions between resident skin cells, infiltrating cells, and vascular tissue. Irritants induce vasodilation, increase of vascular permeability, and inflammatory cell influx from the blood into the surrounding tissues, resulting in erythema and oedema (Scheinman et al., 2021). The distinction between irritants and allergens’ underlying mechanisms is difficult. Recently, molecular profiling showed a unique transcriptomic signature of irritants and allergens, which showed that only allergens are capable of activating the adaptive immunity (especially CD8^+^ T cells). In contrast, irritants trigger keratinocyte damage followed by a rapid tissue renewal supported by the upregulation of cell metabolism, and peptide crosslinking genes (Lefevre et al., 2021).

BAC is a recognized severe cutaneous irritant product used as an irritancy control in numerous experimental studies (Neves et al., 2008; Byamba et al., 2010). As an example, the irritancy potential of BAC and BE was proven in a three-dimensional cultured skin model. BAC and BE revealed a strong skin cytotoxicity by MTT assay and histological skin damage (Nagasawa et al., 2002). BAC, and CKC (cetalkonium chlorure) in a lesser extent, also exhibit a high irritancy potential on eyes, as proven on an *ex vivo* rabbit corneas system (Dutescu et al., 2020).

The damage of the skin barrier is one of the characteristics of irritants. Dodecyltrimethylammonium bromide (DTAB, HTA-type) was assessed for its irritancy potential on human volunteers on their forearms daily for 8 days. The authors showed that DTAB increased transepidermal water loss, and erythema after 4 days of application on forearms. Skin dryness was induced in the chronic exposure model (Wilhelm et al., 1994).

It has been speculated that the irritancy potential of BAC could be attributed to its ROS-inducing capabilities (Rogov et al., 2020). Indeed, BAC induces the release of ROS in monocyte-derived dendritic cells (MoDC) (Byamba et al., 2010) and in HaCAT cells (Kim et al., 2012). A study on the origin of BAC-induced ROS in HaCaT cells showed that ROS were produced via the nicotinamide adenine dinucleotide phosphate (NADPH) oxidase pathway exclusively (Kim et al., 2012). However, Rogov et al. (2020) showed that BAC was able to induce mitochondrial dysfunction in rat liver mitochondria with a decrease in adenosine triphosphate (ATP) synthesis and H_2_O_2_ production.

In addition, MoDCs release pro-inflammatory cytokines such as TNF-α and HaCaT cells release IL-1 α in response to BAC (Byamba et al., 2010; Kim et al., 2012). IL-6 was also shown to be increased in mice model of irritant CD to BAC. In IL-6 receptor deficient mice, BAC induced higher epidermal hyperplasia and inflammatory monocyte influx into lesioned skin but lower numbers of resident macrophages revealing the modulating role of IL-6 in the resolution of inflammation (Frempah et al., 2019). Concerning the eye irritancy, BAC was shown to induce high concentrations of IL-1 and TNF-α by conjunctival and corneal epithelial cells (Epstein et al., 2009).

Another proposed mechanism is the activation of the arachidonic acid (AA) pathway. Indeed, QAC is rapidly incorporated into cell membrane phospholipids which induces its disintegration. It has been reported that irritants could induce the release of AA from the cell membrane of fibroblasts and keratinocytes, leading to the release of other inflammatory mediators. BAC was shown to induce high levels of AA from murine fibroblasts, and the production of prostaglandin E2 thus participating to the pro-inflammatory environment (Moreno, 2000).

Using animal models, the irritancy potential can be studied in mice by measuring the ear swelling after application of a product topically in the dorsal face of each ear. This methodology was used by Shane et al. (2017) which proved the irritant property of DDAB (dimethyldidecylammonium bromide) on Balb/c mice even at low concentration (0.0625%–2%). The same study was done with DDAC (Anderson et al., 2016) in which DDAC induced significant irritancy (0.5 and 1%) (Figure 2A1).

The main studies about the irritant mechanism of QAC are displayed in Table 2.

**TABLE 2:** Sum-up of the main studies about QAC immunotoxicological effects.

Mechanism		Substances	Reference study	Study design
Irritancy	Irritant-induced asthma	QAC (BAC, HTA, CPC, DDAC)	Larsen et al. (2012)	Inhalation challenge in mice
		BAC	Choi et al. (2020)	Inhalation challenge in rats
			Swiercz et al. (2008)	
		DDAC	Ohnuma et al. (2011)	Intratracheal instillation in mice
			(2010)	
		CPC	Kim et al. (2021)	*In vitro* study on A549 cells and intratracheal instillation in rats
		BAC, CPC	Kanno et al. (2020)	*In vitro* study on A549 cells
		DDAC	Kwon et al. (2014)	*In vitro* study on lung epithelial cells
	Irritant-induced dermatitis	BAC, BE	Nagasawa et al. (2002)	*In vitro* study on a three-dimensional cultured skin model
		BAC, CKC	Dutescu et al. (2020)	*Ex vivo* study on rabbit corneas
		DTAB	Wilhelm et al. (1994)	Human study by cutaneous application on the forearm
		BAC	Byamba et al. (2010)	*In vitro* study on MoDC
		BAC	Kim et al. (2012)	*In vitro* study on HaCAT cells
		BAC	Epstein et al. (2009)	*In vitro* study on conjunctival and corneal epithelial cells
Sensitization	Sensitizer-induced asthma	QAC	Preller et al. (1996)	Cross-sectional study of pig farmers
		QAC	Bellier et al. (2015)	SIC in asthmatic patients
		QAC	Migueres et al. (2021)	SIC and study of the sputum of occupational asthmatic patients
	Sensitizer-induced contact dermatitis	DDAC	Anderson et al. (2016)	LLNA in mice
			Shane et al. (2019)	
		DDAB	Shane et al. (2017)	LLNA in mice
		DDAC, BAC	Anderson et al. (2020)	LLNA in mice
Adjuvanticity		QAC	Preller et al. (1996)	Cross-sectional study of pig farmers using QAC
		DDA	Klinguer et al. (2001)	Study of the adjuvanticity using respiratory syncytial virus-sensitizing mice model
			Klinguer-Hamour et al. (2002)	
		QAC	Gall, (1966)	Study of the adjuvanticity using diphteria toxin-sensitizing guinea pig model
		QAC, BAC	Larsen et al. (2004b)	Study of the adjuvanticity using OVA-sensitizing mice model
			Larsen et al. (2004a)	
		BAC	Sadakane and Ichinose, (2015)	Study of cutaneous applied BAC in a dust mite induced-dermatitis model

### 5.2 Sensitizing potential

A sensitizer is defined as an agent that induces a specific immunologic response. Commonly, sensitizers are high molecular weight agents (>10 kDa, usually a protein or a glycopeptide), which are recognized by antigen presenting cells and presented to T lymphocytes. This results to a Th2 response when the cytokines produced are IL-4, IL-5 and IL-13, with the synthesis of IgE antibodies, or rather to a Th1 response, when the cytokines are IFN-γ and IL-12, with a cellular response involving CD8^+^ T cells (Deenick et al., 2011).

According to this theory, QAC which are low molecular weight (LMW) agents should act as haptens, meaning that they should bind to proteins to induce the sensitization step. Usually, sensitizers exhibit highly reactive moieties that can induce protein binding. Depending on the exposure route, the available carrier proteins are different, which implies that a dermal sensitizer is not necessarily a pulmonary sensitizer or conversely. However, BAC was not able to bind covalently to human serum albumin (HSA) under any conditions studied (Aleksic et al., 2007). The same result was found for DDAC in a direct peptide reactivity assay (DPRA) assay (Lee et al., 2019).

#### 5.2.1 Sensitizer-induced asthma due to quaternary ammonium compounds exposure

Sensitizer-induced asthma is generally characterized by the activation of Th2 polarized T cells, with an allergen-specific IgE production and a high eosinophilic count in the sputum (Hammad and Lambrecht, 2021). The mechanisms of sensitizer-induced asthma due to LMW agents is still poorly understood (Maestrelli et al., 2009). However, QAC-specific IgE has been reported in patients suffering from asthma after QAC exposure. Indeed, in the study of Preller et al., QAC-specific IgE were found in 2/40 pig farmers. Even if this very low incidence did not support a role for those antibodies in the apparition of respiratory symptoms, this result showed that QAC could induce a specific immune response (Preller et al., 1996). Moreover, SIC, which are recognized as the diagnostic tool to assess airway responsiveness to a sensitizing molecule, have been performed and found positive in patients suffering from asthma after QAC exposure (Bellier et al., 2015). In a recent retrospective cohort of occupational asthma eosinophilic infiltration of the sputum was found higher in the patients exposed to QAC than in patients exposed to other LMW agents (Migueres et al., 2021). After a SIC to QAC, there were more eosinophils in the patients’ sputum than after a SIC to other LMW agents (Migueres et al., 2021). Those elements plead in favor of a sensitizing mechanism by at least some QAC. However, to our knowledge, animal studies of sensitizer-induced asthma due to QAC exposure are lacking, so the underlying mechanisms are still largely unknown (Figure 2B2).

#### 5.2.2 Sensitizer-induced dermatitis due to quaternary ammonium compounds exposure

Several mice studies have been conducted to understand QAC dermal exposure mechanisms. Anderson et al. (2016) performed a local lymph node assay (LLNA) with DDAC at concentrations ranging from 0.0625% to 1% and found a dose-dependent increase in lymphocyte proliferation. They did not find any IgE production in mice but a significant and dose-responsive increase in the absolute number of B cells, CD4^+^ T cells, CD8^+^ T cells, and dendritic cells in the draining lymph node (DLN), along with a significant increase in the percentage of B cells at day 10 following 4 days of exposure. They also found a significant and dose-responsive increase in the number of activated CD44^+^ CD4^+^ and CD8^+^ T cells and CD86^+^ B cells and dendritic cells following DDAC application, thus classifying DDAC as a T cell-mediated/Th1 sensitizer (Anderson et al., 2016). The same laboratory evaluated the impact of DDAB on dermal exposure. Performing an LLNA assay, they found a dose-dependent increase in lymphocyte proliferation. Unlike DDAC, DDAB induced IgE production as evaluated by phenotypic analysis of DLN B cells and measurement of serum total IgE. The transcription of DLN IL-4, IL-10, and OX40L, and ear TSLP (thymic stromal lymphopoietin) genes were upregulated following 4 and 14 days of exposure (Shane et al., 2017). Thereafter, they studied the potential involvement of type 2 innate lymphoid cell (ILC2) following DDAC dermal exposure (Shane et al., 2019). They showed that DDAC could induce a rapid increase in TSLP expression locally, and correspondingly, dermal ILC2 were activated 24 h after DDAC exposure. This activation led to the release of the Th-2 cytokines IL-4 and IL-5 in the skin and the conclusion that DDAC induced a mixed-type allergic response (Shane et al., 2019). Similarly, dermal exposure of DDAC and BAC were found to induce the upregulation of dermal and DLN IL-4 in mice (Anderson et al., 2020) (Figure 2A2).

The main studies about the sensitizing mechanism of QAC are displayed in Table 2.

### 5.3 Distinguishing irritant and sensitizing properties of quaternary ammonium compounds

As already said earlier, distinguishing irritant and sensitizing properties can be difficult as they are clinically very similar. When using patch testing, which is thought to detect sensitizers, care must be taken when interpreting the results. Irritants give inconsistent reactions in patch test, with “decrescendo” reactions (doubtful early and negative at day 4), while sensitizers induce erythema, papulation, and vesicles, with a “crescendo” reaction. However, BAC sensitizing properties are very controverted as the interpretation of an irritant agent in a patch test can be complicated (Basketter et al., 2004; Uter et al., 2008; Wentworth et al., 2016; Isaac and Scheinman, 2017).

To distinguish irritants and sensitizers, some tests like the LLNA have been proposed. However, some irritants induce a low-level proliferation in the LLNA, that may be differentiated from sensitizers by assessing the B220 + cells or IgG+/IgM+ B cells in the DLN (Gerberick et al., 2002). It was also suggested that the up-regulation of CD86 and HLA-DR on MoDC could differentiate allergens and irritants (Byamba et al., 2010). In this study, BAC was considered as an irritant, given that it was unable to induce the upregulation of those two receptors on the MoDC surface. Moreover, the THP-1 cell line based h-CLAT (human cell line activation test) assay can also be used in this regard (Sakaguchi et al., 2009). According to this test, DDAC was classified as a sensitizer (Lee et al., 2019).

### 5.4 Adjuvant effect of quaternary ammonium compounds

QAC have also been considered as adjuvants in several studies. Adjuvants are defined as substances that enhance the immune response to antigens (Figure 2C).

After the study of Vogelzang, which showed an association between the exposure to QAC in pig farmers and mild bronchial responsiveness (Vogelzang et al., 1997), the same population was explored for IgE sensitization. It was shown that atopic sensitization (presence of specific IgE to common allergens such as dust mites, pollens, or cat) was more frequent among farmers using QAC. This underlines the possible adjuvant effect of these compounds, given that exposure to QAC or sensitization to common allergen alone were not linked to respiratory symptoms (Preller et al., 1996). This potential adjuvant effect of QAC had already been suggested in the literature, for example, using dimethyldioctadecylammonium (DDA) as an adjuvant in vaccines. This compound increased the immunogenicity of various antigens such as toxins, viruses, bacteria, etc. (Hilgers and Snippe, 1992). This effect did not seem to be dependent of the route of administration as it was observed with subcutaneous (Hilgers and Snippe, 1992), intramuscular (Klinguer-Hamour et al., 2002), or intranasal administration (Klinguer et al., 2001). The underlying mechanisms of this effect are not entirely known to date. However, it was shown that the adjuvant effect could be linked to the structure of the QAC in a model of immunization by diphtheria toxoid in guinea pigs. Where a long alkyl chain was absent, no adjuvant effect was observed, while for the compounds with at least one alkyl chain, a chain length-dependent effect was seen. Moreover, the presence of a benzyl group seemed to lower the activity (Gall, 1966). Using a mice model, Larsen et al. (2004a), Larsen et al. (2004b) investigated if the adjuvant effect of QAC was either a Th-1 or a Th-2 response. In this study, BAC increased IgE and IgG1 after one and/or two booster injections, and surprisingly, the lowest concentrations of 0.1 and 1 µg showed the most potent adjuvant effect (Larsen et al., 2004b). These results are in line with a Th-2 adjuvant response of BAC. However, Gall et al. did not find any adjuvant effect of BAC in their guinea pig model (Gall, 1966). The role of BAC has also been investigated in an atopic dermatitis model. In this study, mice were injected with dust mite allergen to induce the skin lesions, and BAC was then applied to the ear envelope. The results showed that BAC worsened the atopic dermatitis skin lesions, and increased the total IgE level, and the dust-mite specific IgG1 production. Interestingly, BAC resulted in higher infiltration and degranulation of eosinophils and mastocytes in the subcutaneous ear tissue and increased the production of inflammatory cytokines in the ear tissue (Sadakane and Ichinose, 2015). Other QAC were also studied [TEA (tetraethylammonium chloride), DDA, CPC, and HTA] by the Danish researchers of the national institute of occupational health in the same model of mice immunized with ovalbumin (OVA) alone or with QAC. They found an adjuvant effect of DDA, as expected, for the IgE, IgG1, and IgG2a levels, at one and two boosters. Concerning CPC, they found a dose-dependent effect of CPC on the IgE level after one booster at the lowest concentrations (0.1 and 1 g), but not at the highest concentrations, and a suppressive effect on the IgE level and also on the IgG1 level after two boosters, for all the concentrations. TEA chloride did not show any adjuvant effect alone but potentiated the effect of DDA when administered together, while HTA showed a suppressive effect on the IgE and IgG1 level after one and/or two boosters (Larsen et al., 2004a). According to those results, a structure-activity relationship was proposed regarding the presence of a long alkyl chain or an aromatic group in the QAC: no or one long alkyl chain-no aromatic group <<< two long alkyl chains <1 long alkyl and one aromatic group (Nielsen et al., 2007).

The main studies about the adjuvant mechanism of QAC are displayed in Table 2.

### 5.5 The link between quaternary ammonium compounds and neuromuscular blocking agents-induced anaphylaxis

Neuromuscular blocking agents are among the most incriminated drugs in perioperative anaphylaxis, leading to increased morbidity and mortality (Mertes et al., 2019). Those reactions occur during the first injection in a significant proportion of patients (Harper et al., 2009). Some characteristics of NMBA could explain this phenomenon (direct histamine release, MRGPRX2 activation, or IgE bridging without carrier protein for example) (Didier et al., 1987; Spoerl et al., 2017). However, the prevalence rate of NMBA anaphylaxis shows a marked geographical variation. For example, the incidence is estimated at 184 per million anaesthesia in France (reaching 251 per million for women) (Mertes et al., 2011). In contrast, the incidence seems less frequent in Sweden (Florvaag et al., 2005), Denmark (Garvey et al., 2001) and in the United States (Gonzalez-Estrada et al., 2021). This suggests that environmental factors might influence the risk of anaphylactic reaction during anaesthesia. Baldo and Fisher showed that the immunogenic properties of NMBA could be attributed to the QA epitope, a pattern shared by all NMBA (Baldo and Fisher, 1983a; Baldo et al., 2009). Moreover, QA-specific IgE are frequently found in patients having experienced NMBA anaphylaxis (Dybendal et al., 2003). Epidemiological studies in Scandinavian countries found that NMBA anaphylaxis were much more common in Norway than in Sweden and that the main difference regarding QAC exposure in those two countries could be attributed to the consumption of pholcodine, used as a cough syrup, which contains a tertiary amine (Florvaag et al., 2005).

Regarding such observations, pholcodine was withdrawn in Norway, with an ensuing decrease of anaphylaxis incidence, confirming that pholcodine might influence NMBA anaphylaxis, even if the underlying mechanisms are largely unknown (de Pater et al., 2017). Sharing the same quaternary epitope as NMBA, QAC have also been suggested as potential sensitizer of NMBA. Therefore, the use of cosmetics has been proposed as an explanation for the high incidence of NMBA anaphylaxis in women (Mertes et al., 2011). In this context, cross-reaction between suxamethonium and QAC has been found using IgE inhibition assay with BAC, quaternium-52, CTAB, hexadecyltrimethylammonium chloride, and ditallow-dimethylammonium chloride. Four of those five QAC were also found to induce basophil degranulation in a suxamethonium allergic patient (Weston and Assem, 1994). In addition, a higher frequency of QAC-specific IgE was found in hairdressers, particularly exposed to QAC-containing products, than in bakers much less exposed. Those specific IgE were cross-reactive for suxamethonium, BAC, and PQ10 (polyquaternium-10) (Dong et al., 2013).

## 6 Discussion and conclusion

QAC are therefore ubiquitous products, and their use is very frequent. Numerous proofs of their irritant, sensitizing and adjuvant properties have been reported, but all the underlying mechanisms are still unknown.

It appears that all the QAC do not exhibit the same effect on the immune system. Regarding the irritancy and the adjuvant properties, BAC seems the most potent among all (Larsen et al., 2004a; Larsen et al., 2012), and it is also the most common QAC found in case reports of asthma or CD symptoms. Thus, the reported effects of BAC could be due to its intrinsic properties although the overrepresentation in case reports is probably also related to its wide use. The fact that all QAC do not have the same adjuvant effects highlight an important role for their hydrophobic associated chain in this property. A classification has thus been proposed about the adjuvant effect of QAC, and the potencies were BAC ≈ CPC > DDA in a mice model of OVA sensitization (Larsen et al., 2004a; Nielsen et al., 2007). On the other hand, the sensitizing properties seem to be related to the quaternary ammonium group. Considering the possible sensitization of NMBA by QAC, the pattern responsible for the cross-sensitization is the quaternary ammonium epitope (Baldo and Fisher, 1983a; Baldo and Fisher, 1983b). Moreover, the IgE sensitization to QAC can be demonstrated by measuring the presence of QA-specific IgE. However, how QAC can lead to the production of specific IgE still raises debate. To induce the production of specific IgE, it is likely that QAC should bind to proteins and that those proteins might differ regarding the route of exposure. However, BAC could not bind to HSA (Aleksic et al., 2007) and DDAC could not bind to any peptides in a DPRA assay (Lee et al., 2019). However, HSA can bind drugs non-covalently through its two pockets [binding site I (in subdomain IIA) and II (in subdomain IIIA)] (Ghuman et al., 2005), and also bind fatty acids (Petitpas et al., 2001b). These bindings involve both hydrophobic and polar interactions between the ligand and the pocket and have already been well studied for warfarin (Petitpas et al., 2001a). Given the amphiphilic structure of QAC, this type of binding could also be considered. Alternatively, another mechanism could be involved, as is the case for nickel, which is capable of forming reversible complexes with proteins (Thierse et al., 2005).

Another hypothesis to overcome the inability of QAC to covalently bind to proteins is a presentation by CD1 molecules. Indeed, CD1 molecules are known to present amphiphilic membrane lipids from self or foreign sources. Their antigen binding clefts are well suited to these molecules by binding the hydrophobic tail inside the pocket and leaving the hydrophobic head on the outside (Salio and Cerundolo, 2015). Contact allergens such as benzyl benzoate and benzyl cinnamate (constituents of Balsam of Peru) have been shown to interact with CD1a molecule and induce a T cell response (Nicolai et al., 2020). Given their structure, such a mechanism could be hypothesized for QAC.

To present antigens, antigen-presenting cells (APC) need to get activated with a danger signal. For example, the ability to upregulate co-stimulation receptors on monocytes, such as CD86, has been shown on THP-1 for DDAC (Lee et al., 2019). This means that some QAC could induce the maturation of APC through their innate properties and then be presented by the APC. The sensitization phase could also be potentiated through the effect of an irritant. Indeed, irritants can alter the epithelial skin or lung barrier, which can increase the permeability and facilitate the entry of the sensitizer. This is particularly important because QAC themselves have irritant properties and because QAC are often associated with other antimicrobials in products that have irritant effects. On this last point, it is necessary to study the effect on the immune system of the association of QAC with other chemicals as they are found together in commercial products. For example, the combination of DDAC and ethylene glycol has been studied by intratracheal instillation of mice. The results showed that the association of the two compounds induced amplifying and dose-dependent cytotoxicity and inflammation in BALF of the mice treated, compared to the chemicals alone (Kwon et al., 2016). It is also essential to consider the global exposure sequence and not assess the effect of a group of chemicals without considering previous exposure to other substances. Indeed, the immune system could be skewed by a chemical, and the response to another chemical applied after that could be different. For example, the effect of DDAC has been studied in a mice model with prior exposure to *ortho*-phthalaldehyde, another disinfectant known for its Th-2 sensitizing abilities (Shane et al., 2020). In this model, prior exposure to *ortho*-phthalaldehyde dramatically increased the IgE response to DDAC and the Th-2 skewing, although DDAC alone is known to induce a Th-1 response (Shane et al., 2020). This also implies that the results of epidemiological studies might have been biased with respect to the overall exposure of the individuals studied.

The majority of the immunotoxicological studies on QAC concern aliphatic and aromatic QAC. Despite the huge number of polyquaterniums used in cosmetics, only few studies have been conducted so far to assess their involvement in hypersensitivities. Among them, studies on quaternium-15, -22 and -39 have been published (Becker et al., 2010; Scientific Committee on Consumer Safety, 2011; Johnson et al., 2016), but data are missing for most of polyquaterniums. Currently, 109 polyquaterniums are registered at CosIng, the European Commission for information on cosmetic substances and ingredients, but many without any CAS of EC number (CosIng—Cosmetics—GROWTH—European Commission, 2022).

Recently, QAC levels were assessed in blood samples of healthy volunteers. In this report, the authors found a detectable level of QAC in 80% of the samples. More interestingly, QAC levels were positively correlated with inflammatory cytokines (IL-6, IL-10 and TNF-α), decreased mitochondrial function, and disruption of cholesterol homeostasis in a dose-dependent manner (Hrubec et al., 2020). However, Vincent et al. (2007) could not detect DDAC in air samplings collected indoors in hospital settings. Indeed, QAC are non- or low-volatile substances and do not likely to contaminate the atmosphere. This indicates that the mode of exposure of QAC could rather be due to an aerosolization procedure, considering the pulmonary route.

Pending further studies, and to reduce the possible harmful effects of QAC, they could be either replaced by other cleaning products, such as green products and methods (steam cleaning, ultraviolet light…), which are less associated with dermal and respiratory health hazards (Garza et al., 2015), or changed in their formulation (avoid spray formulation for example to reduce the aerosolization) (Tarlo et al., 2017). Considering the dermal route, wearing gloves could also be a simple action to reduce the effect of QAC, especially for workers handling QAC.

Interestingly, during the COVID-19 pandemic, QAC has been extensively used. It would be interesting to evaluate the sensitization to QAC before and after the pandemic, and to compare the incidence of dermal and bronchial symptoms, but also to assess whether NMBA anaphylaxis increased during this period, knowing that NMBA have been also much more used than usual due to the need of intubation of patients with severe COVID.

To conclude, accumulated proofs exist concerning the implication of QAC in hypersensitivities. To date, the mechanisms underlying their effects on the immune system suggest that they can either be considered as irritant, sensitizer, or adjuvant according to the studies and the molecules. However, to clearly understand the sensitizing potential, further studies are still expected, in particular to unravel the processes leading to antigenic presentation and the possibility to act as haptens.

## Glossary

AA: arachidonic acid
APC: antigen presenting cell
ATP: adenosine triphosphate
BAC: benzalkonium chloride
BALF: bronchial alveolar lavage fluid
BE: benzethonium chloride
CD: contact dermatitis
CKC: cetalkonium chlorure
CPC: cetylpyridinium chloride
CTAB: cetyltrimethylammonium bromide
DDA: dimethyldioctadecylammonium
DDAB: dimethyldidecylammonium bromide
DDAC: dimethyldidecylammonium chloride
DLN: draining lymph node
DPRA: direct peptide reactivity assay
DTAB: dodecyltrimethylammonium bromide
h-CLAT: human cell line activation test
HCW: health-care worker
HSA: human serum albumin
HTA: hexadecyltrimethylammonium chloride
IFNγ: interferon γ
IL: interleukin
ILC: innate lymphoid cell
LLNA: local lymph node assay
LMW: low molecular weight
MCP: monocyte chemoattractant protein
MIP: macrophage inflammatory protein
MoDC: monocyte derived dendritic cell
MRGPRX2: Mas-related G-protein-coupled receptor member X2
NADPH: nicotinamide adenine dinucleotide phosphate
NMBA: neuromuscular blocking agent
OVA: ovalbumin
PQ7: polyquaternium-7
PQ10: polyquaternium-10
QA: quaternary ammonium
QAC: quaternary ammonium compound
RANTES: Regulated upon Activation Normal T cell expressed and secreted
ROS: reactive oxygen species
SIC: specific inhalation challenge
TEA: tetraethylammonium chloride
TNF: tumor necrosis factor
TSLP: thymic stromal lymphopoietin
